# Xeroderma Pigmentosum: *Man Deprived of His Right to Light*

**DOI:** 10.1155/2013/534752

**Published:** 2013-12-29

**Authors:** Subhash Mareddy, Jithendra Reddy, Subhas Babu, Preethi Balan

**Affiliations:** ^1^Department of Oral Medicine and Radiology, New Horizon Dental College & Research Institute, Sakri, Bilaspur, Chattisgarh 495001, India; ^2^Department of Oral medicine and Radiology, S.V.S Institute of Dental Sciences, Mahabubnagar, Andhra Pradesh 509 060, India; ^3^Department of Oral medicine and Radiology, AB Shetty Memorial Institute of Dental Sciences, Mangalore, Karnataka 575018, India

## Abstract

Xeroderma pigmentosum (XP) is a hereditary autosomal recessive disorder characterized by photo hypersensitivity of sun exposed tissues and subsequent several-fold increased risk for malignant changes resulting from impaired ability to repair UV-induced DNA damage. Estimated incidences vary from 1 in 20,000 in Japan to 1 in 250,000 in the USA, and approximately 2.3 per million live births in Western Europe. Diagnosis is made clinically by the presence of unusual sunburns or lentiginosis or onset of cancers at an early age. It is confirmed by cellular tests for defective DNA repair. Although there is no cure for XP as of now, skin problems can be ameliorated with the use of sunscreens, sun avoidance methods, and recurrent tumor excisions. Oral isotretinoin and topical application of 5-fluorouracil to treat actinic keratoses are other therapeutic options. T4N5 and photolyase liposomal lotions are innovations in the therapy of XP. Genetic counselling implicating the effect of consanguineous marriages should be considered in the management of XP patients.

## 1. Introduction

Xeroderma pigmentosum (literally dry pigmented skin) is defined by extreme sensitivity to sunlight, resulting in sunburn, pigment changes in the skin, and a greatly elevated incidence of skin cancers [1]. It is an autosomal recessive disease with the potential of causing more than 1000-fold increase in the frequency of all types of major skin cancers (basal cell cancers, squamous cell cancers, and malignant melanoma) in areas exposed to sunlight compared to normal population [2]. The basic defect underlying the clinical manifestations is a nucleotide excision repair (NER) defect leading to a defective repair of DNA damaged by ultra violet (UV) radiation [3]. Clinical symptoms can initially be observed in the sun exposed areas of the skin and eyes. Atrophy, telangiectasia, skin tumors, and oral carcinomas are other common manifestations [4]. Oral manifestations usually involve the lips and anterior portion of the tongue and include cheilitis, glossal telangiectasia and leukoplakia [2].

The early diagnosis of xeroderma pigmentosum (XP) is not only crucial for the timely implementation of therapeutic regimens in patients suffering from this disease but it is also key to keeping the development of cutaneous cancers to a minimum. The consequences of a missed or late diagnosis of XP can be devastating. For these reasons, to raise awareness regarding the clinical and public health significance of this rare life threatening disease, we review the epidemiology, pathogenesis, and clinical presentation as well as diagnostic and treatment modalities.

## 2. Historical Background

In 1870, Moritz Kaposi first used the term “xeroderma” to characterize the dry, dyspigmented skin that is the first permanent cutaneous change observed in patients with this disease [5]. In 1874, Kaposi described four patients with xeroderma or “parchment skin” in the early textbook of dermatology, which he wrote with Professor Ferdinand Hebra [5, 6].

The first XP case with neurological signs was described by Dr. Albert Neisser and in 1932, DeSanctis and Cacchione helped coin the term “DeSanctis-Cacchione syndrome” to apply to cases of XP with severe neurological deficiency [7]. Historically, the disease was classified as classical XP with only skin abnormalities and the DeSanctis-Cacchione syndrome with skin abnormalities and extreme neurological degeneration [8]. At least eight inherited forms of xeroderma pigmentosum have been identified till date. They are as follows:

1. Xeroderma Pigmentosum, Type A, I, XPA, classical form,
2. Xeroderma Pigmentosum, Type B, II, XPB,
3. Xeroderma Pigmentosum, Type C, III, XPC,
4. Xeroderma Pigmentosum, Type D, IV, XPD,
5. Xeroderma Pigmentosum, Type E, V, XPE,
6. Xeroderma Pigmentosum, Type F, VI, XPF,
7. Xeroderma Pigmentosum, Type G, VII, XPG,
8. Xeroderma Pigmentosum, dominant type.

More than half of all cases in the United States result from mutations in the XPC, ERCC2, or POLH genes. Mutations in the other genes generally account for a smaller percentage of cases. Complementation groups A and F are more common in Japan than in Europe or the United States [9].

## 3. Epidemiology

XP has been found in all continents and across all racial groups. Consistent with autosomal recessive inheritance, males and females are similarly affected. Estimates made in the 1970s suggested an incidence in the USA of 1 in 250,000 [10] and in Japan of 1 in 20,000 [11]. A more recent survey in Western Europe suggests approximately 2.3 per million live births [12]. Anecdotally, the incidence in North Africa and the Middle East, where there is a high level of consanguinity, is substantially higher.

## 4. Pathogenesis

### 4.1. Molecular Events Associated with Nucleotide Excision Repair of UV-Induced DNA Damage

UV irradiation is composed of both the ultraviolet A spectrum (320–400 nm) and the UVB spectrum (280–320 nm), UVB plays a more pivotal role in the etiology of XP [13]. UV irradiation causes two major photoproducts in DNA: cyclobutane pyrimidine dimers (CPD) and (6–4) pyrimidine-pyrimidone photoproducts (6–4PP). These DNA lesions influence cellular death, aging, mutagenesis, and carcinogenesis when they are not fully corrected by the DNA repair machinery of the cells [14]. XP is an autosomal recessive disorder resulting from mutations in any one of eight genes. The products of seven of these genes (XP-A through G) are involved in the repair of ultraviolet induced photoproducts in DNA by the process known as nucleotide excision repair (NER) [15].

NER comprises two subpathways: the global genome NER and the transcription coupled NER [16–18]. Global genome NER recognises and repairs UV-induced DNA lesions in nontranscribed DNA throughout the genome, while transcription-coupled NER is initiated by damaged DNA-induced arrest of transcribing RNA-polymerase II on the transcribed strand of an active gene [18].

The DNA repair pathway of NER is a multistep mechanism comprising recognition of the UV-induced DNA lesion, unwinding of the DNA from around the lesion, and finally resynthesis and ligation [16, 17]. The XPC and XPE proteins are needed to recognise the photoproducts in DNA. XPB and XPD are part of a protein complex TFIIH, which opens up the structure of the DNA around the site of the photoproduct. XPA protein verifies that proteins are in the correct position and then the nucleases XPG and XPF cut the DNA on either side of the damage, so that the damaged section can be removed and replaced with intact DNA [1].

Thus, subjects with XP have molecular defects in cellular DNA repair mechanisms because of mutations in one or more NER XP genes, leading to hypersensitivity to UV radiation. This results in the accumulation of unrepaired UV-induced DNA damage which either promotes cell death contributing to accelerated skin ageing, or promotes cellular transformation resulting in the development of cancer [17, 19, 20]. Indeed, examination of mutations in the p53 gene in tumours from XP patients reveals p53 mutations characteristic of UV exposure in the majority of tumours [21]. The molecular defect also results in increased UV-induced lethality, which varies substantially between individuals. The level of cell killing is less in individuals mutated in the XPC and XPE genes and with some hypomorphic mutations in other XP genes, because of the residual functional DNA repair. These individuals also do not show the sunburn reaction found in other groups. This has led to the suggestion that the extreme sunburn reaction is likely to be a consequence of cell death [1].

### 4.2. Consanguinity

XP is more commonly seen in populations where marriage of close blood relatives is common [8]. This has been reported to varying degrees of up to 92.8% in XP patients in Libya [22]. It has also been reported in studies from Egypt, Pakistan, and Nigeria among some of the countries that have a high incidence of XP [23]. However, a case report by Stephanic Christen et al. showed that the disease did occur in an uncle and a nephew [24].

### 4.3. Drugs and Chemicals

A number of DNA-damaging agents other than UV radiation have been found to yield hypersensitive responses with XP cell [7, 25] (Table 1).

## 5. Clinical Manifestations

The clinical features are dependent on exposure to sunlight, the complementation group, the precise nature of the mutation, and unknown factors. Consequently, there is a huge variation in clinical features. Sunny climates, outdoor living, fair skin, smoking, poor availability of diagnostic facilities, delayed diagnosis, and poor protection from sunlight will exacerbate the cutaneous features, resulting in multiple pigmentation changes, multiple skin cancers, and early death [1].

### 5.1. Skin Changes

**Figure 1:**
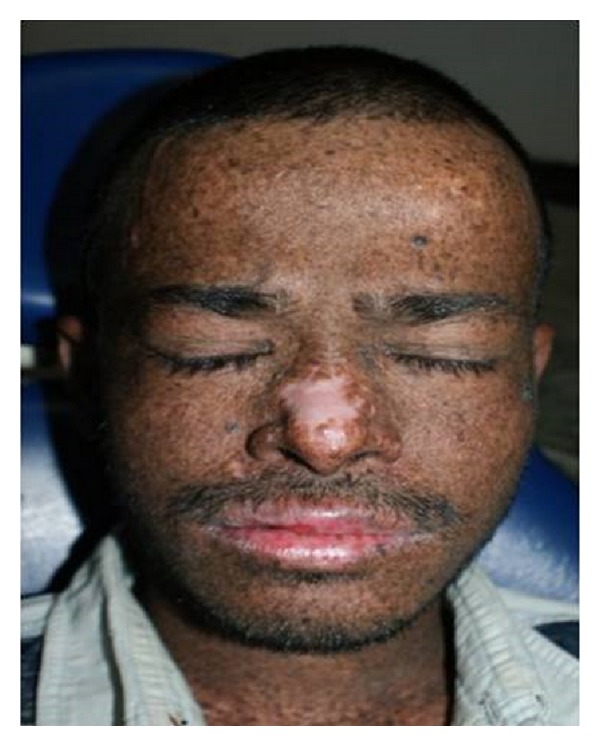
Lentigines (freckle-like pigmentation) on sun-exposed areas of face.

In about 60% of cases, the first sign is extreme sensitivity to sunlight [26], which takes many days or weeks to resolve while the other 40% of cases do not show any sunburn reaction. In these cases, the first manifestation, often by two years of age, is an unusually increased number of lentigines (freckle-like pigmentation) in sun-exposed areas (Figure 1). They are present on the nose, zygoma, and forehead and then appear on the sides of the neck, sparing the area under the chin [1]. Photophobia is often present [27].

**Figure 2:**
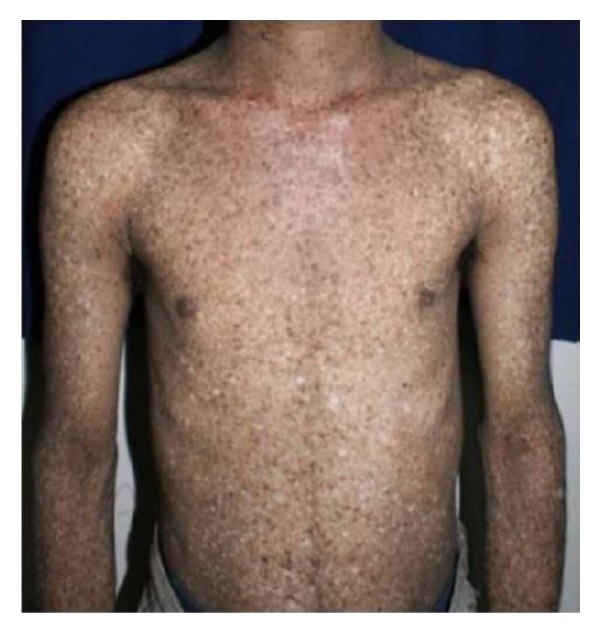
Mottled hyperpigmented and hypopigmented areas giving salt and pepper appearance to skin.

**Figure 3:**
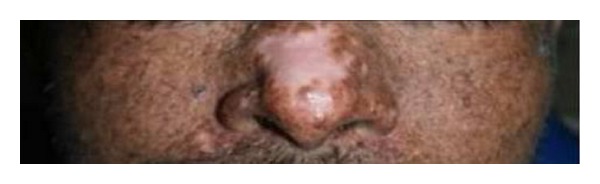
Atrophic, hypopigmented skin of the nose.

In the absence of sun protection, the skin ages, becoming dry, rough, and atrophic. Lentigines increase in number and darken and are difficult to distinguish clinically from the many, flat, pigmented seborrhoeic warts, which also proliferate and become warty. Small, hypopigmented macules are commonly seen amongst the lentigines and giving rise to the characteristic mottled hyperpigmented and hypopigmented appearance known as salt and pepper pattern of skin [7, 18] (Figure 2). Atrophic, hypopigmented patch is often seen on the skin of the nose in these patients (Figure 3). Telangiectasia can be a late feature. Stucco keratoses may be present and are readily distinguishable from solar keratoses.

As all the skin changes are the result of exposure to UV radiation, the severity of these changes is absolutely dependent on the amount of sun-exposure, the Fitzpatrick skin type, and the degree of protection of the skin from sunlight. The effects vary a great deal between individuals.

### 5.2. Skin Cancers

Dark-skinned people usually have a lower incidence of skin cancer compared to light skinned people, most probably owing to the photoprotective properties of melanin. But dark-skinned and light-skinned people with XP, have similar incidences of skin cancer emphasizing the essential role of DNA repair mechanisms even in the presence of protection by melanin [28].

In the absence of rigorous protection from the sun, areas of hyper- and hypopigmentation will result, followed by accelerated photo-ageing, warty lesions, in situ melanocyte and keratinocyte malignancy, and eventually multiple basal cell carcinomas and invasive squamous cell carcinomas and melanomas [1, 26]. Patients with XP under 20 years of age have a greater than 1000-fold increased risk of cutaneous basal cell or squamous cell carcinoma or melanoma [2, 7]. The median age of onset of nonmelanoma skin cancer reported in patients with XP was 8 years, in comparison to 60 years in the general population [29]. It might be expected that the patients with the most severe repair defects would show the most extreme sunburn reactions and the highest incidence of skin cancer. Paradoxically, however, those patients with acute sunburn reactions develop fewer skin cancers than those who do not. This is probably the result of earlier diagnosis of the former group and a disinclination of this group to go out in sunlight without protection.

### 5.3. Ocular Changes

About 40% to 80% of the patients with XP have ocular abnormalities which are caused by UV induced DNA alteration to epithelial cells of the conjunctiva, the cornea, and the eyelid [18]. Photophobia is often present and may be associated with prominent conjunctival injection. Continued sunlight exposure may result in severe keratitis, leading to corneal opacification and vascularization, hyperpigmentation of the eyelids, loss of eyelashes, and in neoplasms (epithelioma, squamous cell carcinoma, and melanoma) [7, 15, 27].

### 5.4. Neurological Symptoms

Approximately 20–30% of the patients have neurological problems with the time of onset varying from the age of two to middle age [26, 28]. It is presumed that as a consequence of defective DNA pathways associated with mutated XP genes, neurons accumulate endogenous genotoxic-induced DNA damage and undergo apoptosis leading to loss of neurons [18]. The neurological abnormalities include isolated hyporeflexia, progressive mental retardation, sensorineural deafness, spasticity, or seizures [30].

Neurological abnormalities are only seen in individuals with defects in complementation groups XPA, XPB, XPD, and XPG. The most common neurological abnormality is a loss of high-frequency hearing while the most severe neurological deficits are seen in DeSanctis-Cacchione syndrome [7, 30]. The incidence for central nervous system tumours (CNS) is also ten times higher than in the normal population. Astrocytomas, medulloblastomas, glioblastomas, and malignant schwannoma are among the CNS tumors [31].

### 5.5. Oral Changes

**Figure 4:**
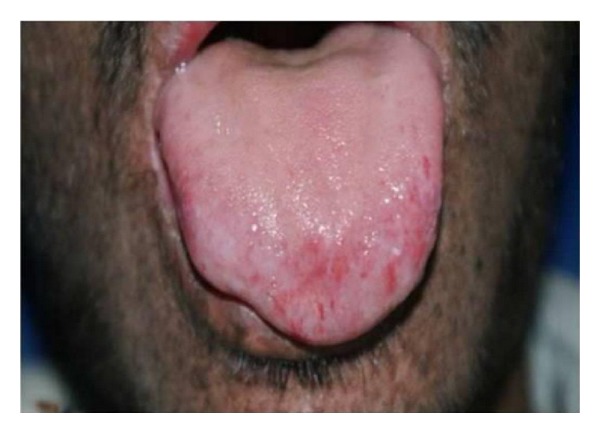
Precancerous lesion affecting the tip of the tongue.

**Figure 5:**
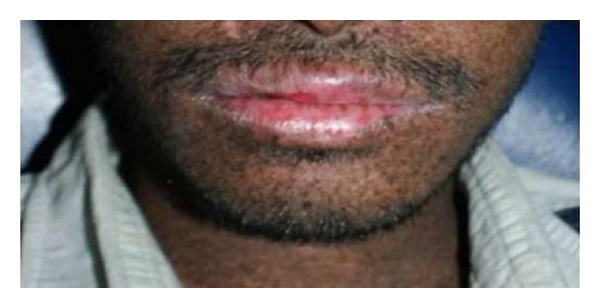
Actinic cheilitis of upper and lower lips.

**Figure 6:**
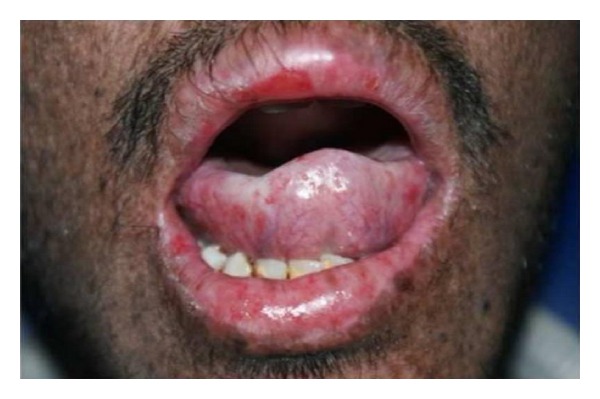
Decreased mouth opening affecting oral hygiene.

Leukoplakia, erythroplakia, and SCC of the tip of the tongue, actinic cheilitis, and SCC of the lips are associated with XP [18]. The precancerous and cancerous lesions of the tip of the tongue, sites seldom affected in the normal population group, are presumed to be induced by UV radiation (Figure 4). This is not a convincing explanation but it is the only one offered [27, 28, 32]. In the general population, SCC most frequently affects the posterolateral and ventral surfaces of the tongue and floor of the mouth of elderly users of tobacco and alcohol and runs an aggressive course. By contrast, XP associated SCC affects the tip of the tongue of persons younger than 20 years of age and runs a slowly progressive course [28]. An actinic cheilitis is a potentially malignant lesion that affects the lower lip of white patients who were frequently exposed to sun (Figure 5). Pain is a consequence of fibrous area, resulting from successive labial plasty, that stretches when the patients opens the mouth for feeding, speaking, breathing, and for oral hygiene performance. Therefore, the patient has poor hygiene habits and consequently, a high rate of dental plaque, caries, and periodontal disease [33] (Figure 6). Cases of chronic desquamative gingivitis, fissured tongue, and keratoacanthoma have been reported [7, 34].

## 6. Diagnosis

### 6.1. Clinical Diagnosis

In most cases, the initial diagnosis of XP is made on the basis of clinical findings and family history. Manifestation of either the extreme sensitivity to UV in those individuals who show this feature, or appearance of lentiginosis on the face at an unusually early age may guide diagnosis [1].

### 6.2. DNA Repair Tests (Classical Diagnosis)

The diagnosis of XP has been made by DNA repair tests such as the measurement of post-UV unscheduled DNA synthesis (UDS), UV survival by colony formation, and the analysis of the recovery of post-UV DNA/RNA synthesis, based on the first discovery by Cleaver in 1968 [3].

#### 6.2.1. Measurement of Unscheduled DNA Synthesis in Cultured Skin Fibroblasts

After DNA damage has been removed, a patch of newly-synthesised DNA replaces the damaged section. Synthesis of this new DNA has some different features from synthesis of DNA during normal replication and the former is therefore referred to as unscheduled DNA synthesis or UDS. Skin fibroblast cultures are established from a 3-4 mm punch biopsy taken from an unexposed area of the skin, such as the upper inner arm or the buttocks. Fibroblasts are UV-irradiated in a Petri dish, and UDS can be measured as incorporation of nucleotides into DNA of the irradiated cells either by autoradiography or liquid scintillation counting [35], or more recently using a fluorescence assay [36]. A reduced level of UDS confirms the diagnosis of XP.

#### 6.2.2. Complementation Test

The classical complementation assay for nucleotide excision repair (NER) defects is based on the analysis of unscheduled DNA synthesis (UDS) in heterodikaryons obtained following the fusion of primary dermal fibroblasts of the patient under study with cells representative of each of the various XP groups. To easily identify the fusion products, the two cell strains used as partners in the fusion are labelled with beads of different size. The two cell strains are classified in the same complementation group if the heterodikaryons, identified as binuclear cells containing beads of different sizes, fail to recover normal UDS levels and remain at the low levels seen in the mononuclear cells. Conversely, the restoration of normal UDS levels in the heterodikaryons indicates that the cell strains used as partners in the fusion have genetically different defects [1, 14].

### 6.3. Molecular Diagnosis

#### 6.3.1. Diagnosis of XPA by PCR

Most Japanese XPA patients can be easily, rapidly, and precisely diagnosed by PCRRFLP using several restriction enzymes. PCR-RFLP is also very useful in early prenatal diagnosis that can be performed with fetal amniotic fluid chorionic villi as a source, in the detection of XPA carriers and might be useful to assess the skin cancer risk in the Japanese general population [14].

The PCR-SSCP (single-strand conformation polymorphism) method, established in 1989, may be useful to find a new mutation in XP patients [37]. It, too, is rapid, easy, and reliable, though it requires the use of radioactive material. The single strand conformation and resulting mobility in the SSCP gel are reflected by the DNA sequence; therefore a mutation of a single base change may be seen as mobility shifts in the gel during electrophoresis [14].

In American and European cases, mutations are detected at different sites in the XPAC gene than in Japanese cases [14].

#### 6.3.2. Diagnosis of XP by Plasmid Host Cell Reactivation (HCR)

Host cell reactivation (HCR) is an easier, more rapid, and more sensitive laboratory assay for the diagnosis of XPB-XPG patients and non-Japanese XPA cases. HCR utilizes an ultraviolet (UV)-treated plasmid containing the sequence of a reporter gene such as chloramphenicol acetyltransferase (CAT) [38]. The plasmid, pRSVcat, is treated with UV and transfected into XP cell lines from patients with cloned XP genes (XPA-XPG, control plasmid). After repair and expression of pRSVcat for 2 days, CAT activity is measured and DNA repair capacity (DRC) is calculated as the percentage of the residual CAT gene expression after repair of damaged DNA compared to undamaged DNA, which is considered as 100%. The recovery of CAT activity is decreased with increased UV doses in all of the cell lines [14].

### 6.4. Prenatal Diagnosis

The DNA repair tests described above can be carried out on chorionic villus-derived cells or on amniocytes in affected families, as can molecular analysis if the mutation in the proband has been identified [1]. The efficacy of preventive measures in X.P. is at least partly dependent upon the early postnatal detection of index cases as exemplified by the history of the family.

## 7. Treatment

**Figure 7:**
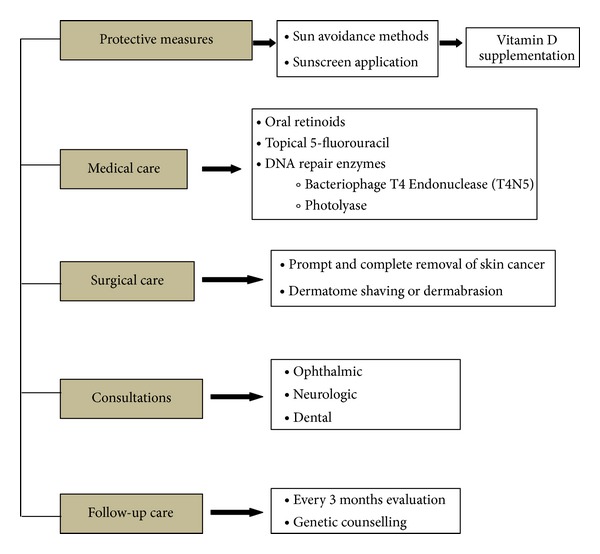
Treatment protocol for xeroderma pigmentosa.

There is no cure for XP. The DNA damage is cumulative and irreversible [7]. Early diagnosis and extensive sun protection have the potential to prevent skin cancers in XP patients and prolong their life expectancy [33] (Figure 7).

### 7.1. Protective Measures

Persons with XP must avoid exposure to any sources of UV light including sunlight, fluorescent, halogen, and mercury-vapour lights [28]. UV-absorbing films and filters can be placed over windows and fluorescent or halogen lamps. Sun protection can be achieved by wearing protective clothing and UV-absorbing eye glasses, with side shields and use high protection factor sunscreens [27]. Rigorous sun protection is likely to result in vitamin D deficiency, so vitamin D supplements should be prescribed [18].

Eye care consists of sunglasses, artificial tears, steroid drops, and bland ointment at night. These are essential components of a prevention program [33].

### 7.2. Medical Care

XP associated cutaneous, ocular, and oral lesions and disorders should be treated as in any other person.

#### 7.2.1. Oral Retinoids

Use of isotretinoin, a vitamin A derivative, has been shown to prevent the appearance of new skin neoplasms in XP patients during oral medication of the drug [39]. It has been associated with toxic effects, such as hypertriglyceridemia, hepatic dysfunction, teratogenicity, and skeletal abnormalities as well as rapid reversal of prophylactic effects upon withdrawal [39].

#### 7.2.2. Topical Therapy

Chemical therapy with 5-fluorouracil may be useful for actinic keratoses. Methyl cellulose or quinodine-containing eye drops, and bland ointment at night constitute correct eye-care [27, 40].

#### 7.2.3. Potential Therapies

*Bacteriophage T4 Endonuclease (T4N5).* Bacteriophage T4 endonuclease V (T4N5), a polypeptide with a molecular weight of 16,500, possesses specific activity against cyclobutane pyrimidine dimmers (CPDs) [41]. Application of T4N5 liposome lotion (which is prepared by mixing T4N5 liposomes into a 1% hydrogel lotion [Lipo Chemical, Inc.]), following UVB-induced damage, has been shown to increase CPD repair at the application site [42]. Thus, it may be a useful tool to prevent the new appearance of actinic keratosis or other skin tumors in XP patients [14, 42].**

*Photolyase*. Photolyase, another enzyme that repairs DNA, is found in fish, reptiles, marsupials, and plants but not in humans. Photolyase holds promise because it binds to CPDs specifically and removes them upon exposure of the complex to light. Wavelengths of 300 to 500 nm convert the pyrimidines back to their monomeric form. This mechanism of light dependent DNA repair, called photoreactivation, has been reported to restore immune competence to UV-irradiated antigen-presenting cells [41, 43].

### 7.3. Surgical Management

#### 7.3.1. Resection

As there is no cure for the genetic disorder XP, the main goal of treatment involves the prompt and complete removal of skin cancers by skin surgeons. In addition to the surgical resection of skin tumors, local injection of skin neoplasms with interferon alpha may be useful for melanoma in situ [44].

#### 7.3.2. Dermatome Shaving or Dermabrasion

Large areas of affected skin in patients with XP have reportedly been treated with dermatome shaving or dermabrasion [45]. Dermabrasion and isotretinoin therapies do not correct the underlying defect (DNA damage) and long-term therapy with dermabrasion, therefore, is neither ideal nor convenient.

### 7.4. Consultations

Regular visits should be paid to the dermatologist for early detection and treatment of all neoplasm as well as for purpose of patient education [7]. Frequent eye examinations by an ophthalmologist are recommended [1]. Neurological evaluation by routine audiometry, measurement of head circumference, assessment of gait and deep tendon reflex testing can serve as a screen for the presence of XP associated neurologic abnormalities. Management of such patients includes the use of hearing aids, physical therapy, occupational therapy and speech therapy [1]. Cases with XP neurodegenerative disorder may have problems with their teeth such as dental caries and should be evaluated periodically by dentists [14].

### 7.5. Follow-Up Programme

Patients should receive follow-up care every 3 months. Follow-up care should be focused on educating the patient and the patient's parents about effective sun protection and early recognition of skin cancer. Genetic counselling should be offered for families at risk.

### 7.6. Gene Therapy

Recently, an experimental gene therapy for XP cells or XP mice has been investigated [46]. This method, currently at research level, offers hope for the future.

## 8. Oral Health Considerations

Clinical examination should be carried out regularly by the dentist for the detection of premalignant or malignant lesions. Furthermore, it is necessary to establish protocols for prophylaxis and topical fluoride application, as well as the use of chlorhexidine digluconate 0.12%, aiming the homeostasis of the oral environment. The use of mouthwashes with high alcohol concentration should be avoided because there is an increased risk of developing oral cancer in these patients [47]. Furthermore, regular dental procedures, such as dental extraction, restoration and rehabilitation become a challenge for the dentist due to difficult access to the oral cavity [33].

The dentist must use a UV light meter to test every light source that the patient could be exposed to during the visit, including overhead lights, dental lamps, viewboxes, fiberoptic lights, computer screens and dental curing units. Any reading above 0 nm/cm^2^ for UV light should contraindicate the use of that unit [4]. Although light-emitting diode curing lights are manufactured to emit a wavelength of only 450 nm, the extent of biological damage from exposure is not adequately understood. For these reasons, restorative materials such as glass ionomers offer a good alternative to resin sealants and composite restorations for treating XP patients [48].

## 9. Outcome

The prognosis varies with the severity of the genetic disorder, the success in avoiding UV light and vigilance of screening as well as extent of any neurological involvement [3]. The most common cause of death was skin cancer (metastatic melanoma or invasive SCC). Despite an early age of skin cancer diagnosis, about 45% live into their 40s and the oldest patient died at the age of 73 years [49]. Patients with xeroderma pigmentosum and their families face many challenges in daily living. Constant education and reminding of the need to protect oneself from sunlight are paramount for a relatively normal lifespan.

## 10. Conclusion

The extensive ultraviolet radiation induced skin and eye damage in patients with xeroderma pigmentosa are an evidence of the unawareness of the lethalness of the disease thus contributing to neglect of sun-protection. Currently, there is no cure for XP. In the future, we hope larger clinical trials are conducted to increase the understanding of the aetiology of the disorder and for the development of treatment to alleviate affected patients and help them improve their quality of life and life expectancy. After all, “*Every child has the right to light*.”

## Figures and Tables

**Table 1:** Exogenous DNA damaging agents.

Physical agents	Chemical agents
(1) UV radiation (sunlight)	(1) Plant toxins—Aflatoxin
(2) Ionising radiation	(2) Alkylating agents
X-rays	(3) Nitroquinoline oxide derivatives
Gamma rays	(4) Acetylaminofluorene derivatives
(3) Smoke	(5) Phenanthrene derivatives
